# Clustering Consumers Based on Trust, Confidence and Giving Behaviour: Data-Driven Model Building for Charitable Involvement in the Australian Not-For-Profit Sector

**DOI:** 10.1371/journal.pone.0122133

**Published:** 2015-04-07

**Authors:** Natalie Jane de Vries, Rodrigo Reis, Pablo Moscato

**Affiliations:** 1 Centre for Bioinformatics, Biomarker Discovery & Information-Based Medicine, The University of Newcastle, Callaghan, New South Wales, Australia; 2 Faculdade de Medicina de Ribeirao Preto, Universidade de São Paulo, São Paulo, Brazil; East China University of Science and Technology, CHINA

## Abstract

Organisations in the Not-for-Profit and charity sector face increasing competition to win time, money and efforts from a common donor base. Consequently, these organisations need to be more proactive than ever. The increased level of communications between individuals and organisations today, heightens the need for investigating the drivers of charitable giving and understanding the various consumer groups, or donor segments, within a population. It is contended that `trust' is the cornerstone of the not-for-profit sector's survival, making it an inevitable topic for research in this context. It has become imperative for charities and not-for-profit organisations to adopt for-profit's research, marketing and targeting strategies. This study provides the not-for-profit sector with an easily-interpretable segmentation method based on a novel unsupervised clustering technique (MST-kNN) followed by a feature saliency method (the CM1 score). A sample of 1,562 respondents from a survey conducted by the Australian Charities and Not-for-profits Commission is analysed to reveal donor segments. Each cluster's most salient features are identified using the CM1 score. Furthermore, symbolic regression modelling is employed to find cluster-specific models to predict `low' or `high' involvement in clusters. The MST-kNN method found seven clusters. Based on their salient features they were labelled as: the `non-institutionalist charities supporters', the `resource allocation critics', the `information-seeking financial sceptics', the `non-questioning charity supporters', the `non-trusting sceptics', the `charity management believers' and the `institutionalist charity believers'. Each cluster exhibits their own characteristics as well as different drivers of `involvement'. The method in this study provides the not-for-profit sector with a guideline for clustering, segmenting, understanding and potentially targeting their donor base better. If charities and not-for-profit organisations adopt these strategies, they will be more successful in today's competitive environment.

## Introduction

Far from being a calm and slow-paced segment of the tertiary economic sector, charities and not-for-profit organisations need to be more proactive than ever. They are demanded to adapt to modern technologies and to be more efficient in a highly-competitive environment in which they are increasingly competing for donors’ time, trust, confidence, involvement and charitable giving. This new type of behaviour for institutions that were previously characterised as “less agressive” in their marketing strategies is progressively imitating the activities of regular for-profit organisations as competition amongst charities becomes more fierce [1].

Some characteristics of charitable giving may be highly dependent of the particular country or social group under consideration. For instance, researchers have previously argued that Australian consumers in particular perceive government’s support for charities to be quite high, consequently impacting on their perception of charities’ need for individual support [2]. Furthermore, ‘trust’ is a defining concept when it comes to individual charitable giving and it could even be stated that ‘trust’ constitutes the very foundation on which voluntary institutions are built [3]. It is therefore an interesting subject to investigate Australian consumers’ support in terms of donating and volunteering behaviour related to their trust and confidence in charities.

This study aims to investigate these issues in the scenario of Australian consumers’ attitudes towards and trust and confidence in charities. The methodology proposed to segment the population in particular clusters of more homogeneous attitudes is quite general and can easily be employed to analyse other datasets. From a marketing perspective, our study addresses the issue of charities and not-for-profits’ need to behave more competitively among themselves. The purpose of this study is to identify if it is possible to obtain more homogeneous groups of attitudes and consumer behaviours towards charities. We seek to identify whether there are clear patterns among respondents who are highly similar to each other in terms of their behaviours and attitudes towards charities. Also, a further analysis of which characteristics are most representative of each cluster is provided. We note that clustering of the market, or; *market segmentation*, is common practice in private for-profit organisations in order to better understand the drivers of their consumers, create targeted marketing and advertisement strategies that are aware of these differences and to select those consumer groups worth pursuing. We bring a novel method that provides advanced clustering strategies into the context of Not-For-Profit (NFP) organisations and thus cluster a population of potential donors based on their trust and confidence in, and their behaviours towards the existing spectrum of charities in a particular country.

The paper is organised as follows. First, a background to the study including a brief review of the literature is provided, followed by Materials and Methods which introduces the dataset used, the methods employed in its processing and the methodology utilised in this study followed by the Results. Finally, the Discussion section discusses some of these results with implications for managers of charities and provides final remarks for future research.

## Background

Charities all over the world are facing increasing competition from one another and other NFP organisations for people’s time, money and efforts. It has long been argued that not-for-profits are following in for-profit organisations’ footsteps and that they are attempting to adopt more targeted strategies for finding their most likely donor base [1, 4, 5]. This trend however, has become even more important in the modern interconnected world where consumers’ time is sparser, globalisation has brought consumers and institutions together globally as well as increased competition between institutions for consumer’s time and money [6]. As stated by Polonsky, Shelley and Voola [2], charities are utilising a range of marketing activities in order to simply maintain private donations.

One such marketing activity is that of market segmentation [1]. Market segmentation is a strategy well-known to private industry marketers being introduced several decades ago [7] and has since been widely utilised in for-profit organisations. As in the case for businesses, effective market segmentation should allow charities and NFP’s to customise the message content of their appeals and campaigns to distinct groups of prospective donors [1]. In doing so, identifying and selectively targeting the most likely individuals, or groups of giving individuals, would optimise the ratio of successful approaches to total number of approaches [8]. However, as stated by Srnka, Grohs and Eckler [8], although the “who” and “how” questions are important in segmenting, it is the more detailed factors such as behavioural variables that provide a better segmentation approach. Furthermore, for the charity and NFP sector, public trust and confidence are at the pinnacle of its existence and of extreme importance to its success and longevity. This is why in the case of segmenting the market for potential charitable donors, not only their behaviour is important, but also their trust, and confidence in a charitable organisation.

In this context, it is important to note that donating and/or volunteering behaviour are not substitutable to trust or confidence in an organisation. As explained by Bekkers and Bowman [9], some individuals engage in volunteering activities even with the lowest levels of charitable confidence. This means that some individuals may have low levels of trust and confidence in charities but are still willing to volunteer and donate, and oppositely, there are individuals who do trust charities but neither engage in volunteering activities or donating financially. This complex relationship between trust and giving behaviour has been examined empirically in a causal path model where trust and ‘commitment’ to the charity are found to be positively linked to donor giving behaviour [10]. This means that when investigating a consumer group in terms of donating behaviour, trust and confidence in charities form part of the major factors. Furthermore, this extant literature highlights the complexity of consumers’ trust in, and confidence of charities and NFP organisations and the need for further empirical examination.

Another important aspect in the context of donor behaviour and charities’ operations to note, is that of consumers’ perceived wastefulness of charities’ resources. A recent study has found that avoiding donor’s funds going to overheads (i.e. administrative and advertising costs) and enlightening consumers of this overhead aversion increases donations significantly [11]. Specifically, Gneezy et al. [11] find that when informing potential donors that seed funding has already been obtained (from philanthropists or companies), donations rates significantly increase by an astonishing 80%. Interestingly, the study by the Australian Charities and Not-for-Profits Commission (ACNC) of which the data is used in this study, included many financial-focused questions asking respondents about their perceived wastefulness as well as the types of information they value. Furthermore, the link between donor’s trust in charities and their perceptions on how charities manage resources and donations is covered in this study making it an interesting case to test the findings by Gneezy et al. [11].

It is for these reasons that this study aims at segmenting the Australian consumer market in terms of trust and confidence in, and donating behaviour towards charities and not-for-profit organisations. Private donations form an issue for charities and not-for-profit institutions in Australia in particular as Australian donors perceive that government support of charities is high and thus charities are perceived to be less likely to need individual support [2]. This provides one of the many reasons it has become inevitable for Australian charities to embrace more advanced strategic marketing activities. However, due to the philanthropic nature of charities and not-for-profits, funds are limited; particularly funds for marketing research and administrative costs. In this sense, we believe that this research adds a valuable contribution to the literature by utilising advanced research techniques in a not-for-profit context helping charities and NFP organisations to better understand the Australian donor market.

## Materials and Methods

### Data Set

The data employed in this study consists of a set of responses from a quantitative survey conducted on behalf of the Australian Charities and Not-for-profits Commission (ACNC) by ChantLink in 2013. The data as well as the survey and report are publicly available online and are maintained by the Office of the Australian Government *http://data.gov.au/dataset/trust-and-confidence*. This survey collected information about levels of trust and confidence in charities and factors that may affect these levels. It also collected information about awareness and support for a national regulator of charities and interest in a public register of charities.

The survey was conducted online between 22 and 29 April 2013 and obtained 1,624 complete responses (including a pilot phase of 60 responses). For the purpose of this study we omitted the pilot phase responses leaving 1,562 responses for our clustering experiments. Furthermore, in this data set, due to the design of the online questionnaire, not all participants responded to every single question. Depending on their responses, individuals were sent to a different section and were forced to skip several questions. Therefore, in order to obtain clusters without the need to use mechanisms to impute missing data for a large proportion of the sample, the complete data set of *n = 1,562* respondents was checked and only those questions answered by all individuals are used in the clustering process. As a result, we have 1,562 responses to 43 different questions which form the basis to compute similarities among the respondents for the clustering method. In other words, we have a 1,562×43 matrix of our data. This matrix provides the basis for the computation of a similarity (or distance) measure needed for the clustering process which is described further below.

Some of the remaining questions, which, for instance relate to the respondents’ demographic information, are subsequently used to highlight possible differentiating characteristics of the clusters. We do not include this information in the clustering method, since segmenting a market based on consumers’ behaviours, trust and confidence in charities is our primary goal and it provides a more detailed cohort analysis than segmenting based on demographic information alone.

### MST-kNN Clustering Method

In order to segment the sample of Australian donors, a novel and highly scalable clustering method is employed. The method aims at finding groups of related respondents that share similar characteristics. These similar characteristics are measured using a particular distance or similarity (correlation) metric. The selection of this distance or correlation metric is a significant step as it defines when two respondents are going to be in the same group; therefore whether they are ‘closely related’ or not. In order to analyse the data and to find meaningful groups we use the unsupervised graph-based clustering algorithm: MST-kNN [12] which has previously been used in bioinformatics and in the analysis of a wide range of studies including; Alzheimer’s Disease and yeast transcriptomic datasets, prostate cancer, computational linguistics, RNA stability and shows great scalability and performance [13–16].

In this study, the Spearman rank correlation coefficient is used to help us define a distance or ‘dissimilarity’ between pairs of respondents. This means that this metric is computed for every possible pair of respondents based on their responses in our 1,562×43 matrix. Consequently, the computed a correlation matrix which is a 1,562 by 1,562 array consisting of the correlation values for every possible pair of respondents. The Spearman rank correlation coefficient is a statistical measure of a monotonic relationship between two different variables. We denote the Spearman correlation with *S*_*xy*_ and the distance metric between two respondents *x* and *y* by the expression (1).

$$\begin{array}{c} d_{xy}=1-S_{xy} \end{array}$$ (1)

A strong Spearman correlation between two variables may suggest that they are related via a monotonic function, alternatively, a strong Pearson correlation, indicates that they are related by a linear function. Since the latter is a more restrictive relationship, we have preferred the use of Spearman rank instead of Pearson as the assumption of linearity has been criticized by the area of computational social sciences by researchers such as Herlocker et al. [17]. Furthermore, since the Spearman correlation does not depend on the assumption of linearity, and it is less sensitive to strong outliers, it is preferable for the purposes of this study [18].

**Fig 1:**
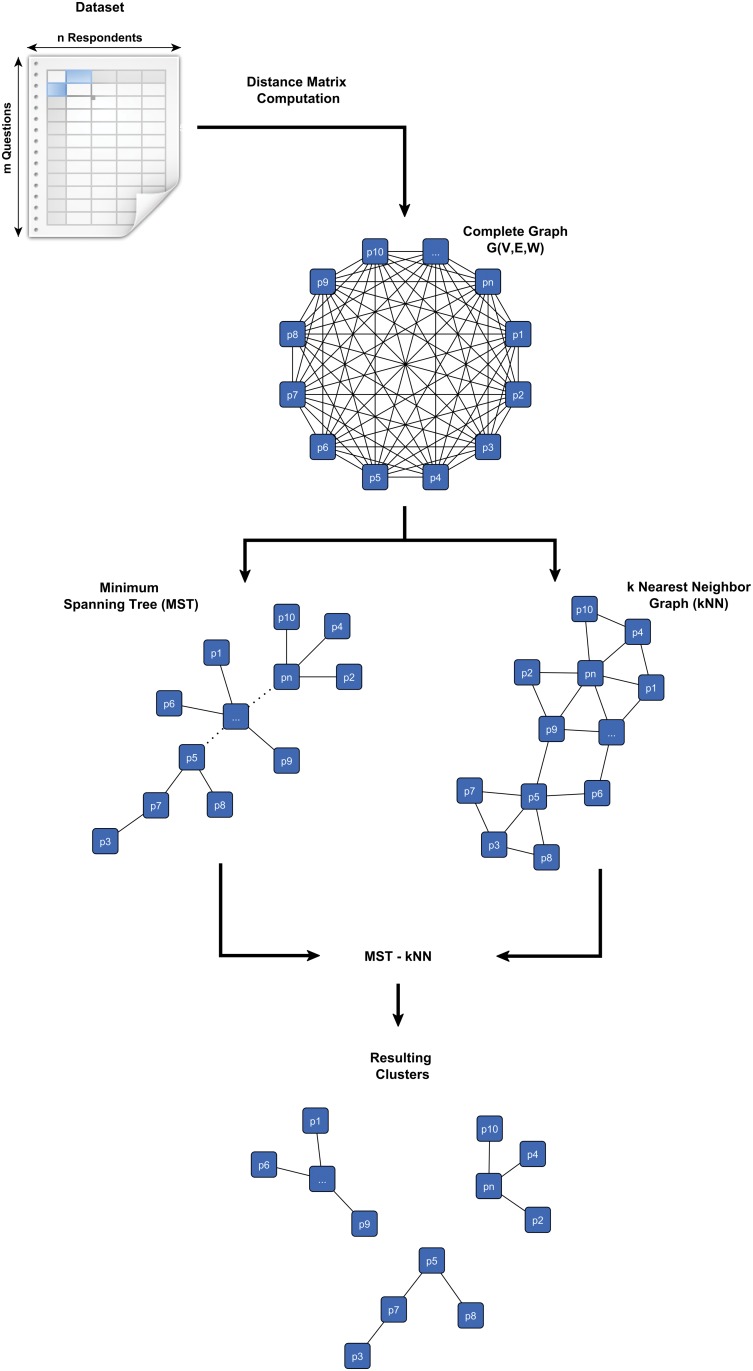
Process of the MST-kNN method. Starting with the complete dataset, a distance matrix is computed which forms the basis for a complete graph. A Minimum Spanning Tree is computed within the complete graph. Then, all edges that are not k-Nearest Neighbors are removed resulting in clusters.

Fig 1 shows the process of our MST-kNN method. It outlines the process of the MST-kNN clustering algorithm. This algorithm is based on the computation of two types of proximity graphs: the Minimum Spanning Tree (MST) and a k-Nearest Neighbor graph (kNN). The algorithm first builds an undirected complete weighted graph *G*(*V*,*E*,*W*), with a vertex *v* ∈ *V* for each of the respondents sets, and an edge *e* ∈ *E* for each of the respondents pair, with the edge’s weight *w* ∈ *W* being the distance (which will be equal to one minus the Spearman rank correlation coefficient between the responses of each respondent). Given *G*, the algorithm computes the Minimum Spanning Tree, that is a graph with no cycles such that the total sum of the distances in the edges of the tree is minimum among all possible trees that connect all vertices (the ‘spanning’ requirement).

A simple undirected graph *G(V,E,W)* is a tree if any two vertices of *V* are connected on unique simple sequence of edges belonging to *E*. This is the outcome of computing the minimum spanning tree. Following this, the algorithm computes a *k*-Nearest Neighbor graph where the vertices are connected if they are reciprocally *k*-nearest neighbors of each other according to a defined value of *k*. The *k*-nearest neighbours are defined as the *kth* most similar vertices according to the measure used (in this case; one minus the Spearman rank correlation between two respondents). The higher the value of *k*, the more densely connected the graph becomes. In the clustering process utilised in this study, we define this value as *k* = 3. This means that any two vertices connected by an edge in the 3-Nearest Neighbor graph are no more apart than the third smallest distance between one of them to all the other vertices.

The edges 3-Nearest Neighbor graph is used to select some edges of the MST for deletion, i.e. two vertices in the MST are not one of the 3-nearest neighbours of each other. If at least one edge is deleted from the MST, this creates a set of trees, rather than one fully connected graph, which partitions the set of respondents in clusters.

Although the use of proximity graphs such as the MST and kNN methods by themselves is already functional and can show different structures in the data, their combination is even more powerful. A significant characteristic of this combination is that it does not require additional information other than the existing inter-pair distances to produce a clustering. This is a great advantage since the structure of clusters in a high-dimensional space (here we have 43 questions of interest) is not always known *a priori* or has either predefined labels or classes among the samples. A further benefit of the MST-kNN algorithm is that it is highly scalable, allowing its application in large data sets involving millions of samples [16]. Due to its characteristics, it provides a powerful novel approach to the analysis of questionnaires and the segmentation of the respondents. It is for these reasons that we employ this method in order to segment the group of respondents into identifiable sub-groups which charities and not-for-profit organisations can subsequently target.

### CM1 Scores

In order to highlight the individual and most clearly identifiable characteristics of each cluster generated by the MST-kNN agglomerative algorithm, a new score introduced in the area of Computational Linguistics [19]; and Bioinformatics [20] was computed. In other words, the use of the CM1 score in this study is to find the most *salient* features for each cluster. As Aviad and Roy [21] explain, in many classification or clustering problems, the user needs to exert considerable effort to interpret the results. It is for this reason that a tool such as the CM1 score, which highlights feature saliency in each cluster, is useful for interpreting and understanding clustering results.

For each of the answers given, we compute its associated CM1 score which gives an indication of the differences in averages of that particular question between the members of a cluster and those who do not belong to that cluster, normalized by the range of the values observed in the individuals outside the cluster. The CM1 scores for a cluster can be split into ‘top’ and ‘bottom’ scores. The ‘top’ scores refer to those that have an average which is greater in a specific cluster than in all the others, while the ‘bottom’ scores refer to those attributes which average is lower in a specific cluster than in the others. For instance, in the previous application of the CM1 scores in a bioinformatics context in [20], the ‘top’ scores represent those genes that are are highly expressed, or ‘upregulated’ in that cluster (or group) and the ‘bottom’ scores represent those genes that are ‘downregulated’ in that particular group in comparison with the outgroup (normalized by a function of the range observed in the outgroup.

CM1 scores are computed using Expression 2. The scores are computed using the difference between the average of samples in a specific cluster, *X*, and all the others, *Y*. This difference is moderated by the range of values observed in members of all the other clusters, *Y*, which has a greater set of samples, instead of the combined standard deviation of the specific cluster, *X*, and all the other clusters together; *Y*. For specific details of this score we refer to a study published by Marsden et al. [19].

$$\begin{array}{c} CM1\left(w,X,Y\right)=\frac{\frac{1}{\left|X\right|}\sum_{x\in X}x_{w}-\frac{1}{\left|Y\right|}\sum_{y\in Y}y_{w}}{1+max_{y\in Y}\{y_{w}\}-min_{y\in Y}\{y_{w}\}} \end{array}$$ (2)

Results of the CM1 score analysis are used in this study to describe the clusters resulting from the MST-kNN algorithm as these variables are the most discriminative of each cluster. Furthermore, the separation and description of the clusters using the CM1 score is investigated in further detail through the use of symbolic regression modeling. By modeling each cluster as the target variable against results from all other clusters and using only those variables that are the ‘top’ and ‘bottom’ features, we are able to further outline, describe and separate between clusters. Details of this method are outlined in the following section.

### Symbolic Regression Modeling

#### Target Variable Selection

To further investigate and gain a greater understanding of each of the clusters, symbolic regression modeling is used in this study. Unlike with “normal regression” approaches, where models are hypothesised and generated to fit the data, symbolic regression discovers the model structure as well as the coefficients within that structure that produce the best fit [22]. This means the models are “driven by the data”. In this study we aim to find data-driven models using this approach for various behaviours towards charities and NFP organisations for each cluster.

This methodology is adopted from research in biological methods where *‘reverse-engineering’* a model from the data is a common and accepted practice [23]. Recently, we adopted this method in another computational social science context in which we reverse-engineered a customer engagement model based on survey data rather than hypothesised relationships as is common practice in marketing studies [24]. Our intention here is to take a similar approach, but rather than finding a model for the whole data set, we try to find a particular model to suit each cluster. The purpose of this is to predict whether a donor is more likely to be highly involved or less involved with charities and not-for-profits based on their trust and confidence in, and attitudes towards charities. As in [24], we use the symbolic regression modeling software *Eureqa*[25], a commercial package which is free for academic use, making the method easier to adopt in future studies.

There are several models that were generated using the *Eureqa* software. As stated, the first experiment aims to find a model that could “describe” the clusters as a function of the most discriminative CM1 scores. *Eureqa* allows to search such classification models by introducing a binary target variable (set to 1 for any sample in the cluster of interest and 0 for all the others). This means that for each cluster we assign the value 1 to the cluster of interest and 0 to all other samples and repeat this for each cluster. Then, we set the cluster of interest as our target variable and let *Eureqa* find the best suited model to describe each cluster. In other words, *Eureqa* attempts to *predict* those variables that characterise whether a respondent is from the cluster of interest or not. In this way we assess the outcomes of the clustering method and confirm the description of the clusters using the CM1 score.

After this, further modeling is done in order to predict *’Involvement Class’* as a function of all attributes relating to trust and confidence in charities, and general knowledge and care about a national charity and not-for-profit registry. The ACNC classed the respondents as either *‘High’* or *’Low’* in involvement based on their answers of donating behaviours. For instance, questions about whether a respondent has previously donated money, how often they donate, whether they volunteer, how often they volunteer or whether they have sponsored a child or an animal are examples of activities which the ACNC classed as ‘High Involvement’. Based on the responses to these questions, the respondents were classified as either *’Low involvement’* or *’High involvement’* which is depicted in the data set as 0 and 1 respectively. This binary variable is then set as the ‘target variable’ in *Eureqa* and a model specific for each cluster is then found using the software.

#### Symbolic Regression Modeling and Optimality Measures

Before modeling the outlined experiments in *Eureqa*, certain user-defined ‘building blocks’ need to be selected [25]. In this study, for each experiment we selected only the *’constant’, ‘integer constant’, ‘introduction of an input variable’, ‘addition’, ‘subtraction’* and *’multiplication’* building blocks. Furthermore, due to the binary nature of our target variables, it is recommended to trial the use of the *logistic function*. This function is used to search for equations that tend to be negative when the output is false ‘0‘, and positive when true ‘1‘. Therefore, we searched for models using the *logistic function* as function of the search, *y* = *logistic*(*f*(*x*)), following the recommendation of the *Eureqa* manufacturers to deal with binary classification problems. No data splitting option was used as all data points are treated equally. Being a binary classification problem, it is natural to evolve models using the Area Under the Curve (AUC) as a fitness function of the evolutionary algorithm which is the search engine of *Eureqa*.

With this sensible selection for a binary classification problem, *Eureqa* runs its evolutionary search procedure to find a model that best fits the data. *Eureqa* uses a Pareto-optimality front as it aims at both minimising the error and the complexity of the models [22]. This process is repeated for each of the clusters after which the results for each cluster are compared to each other. Results of this process are displayed in the following section.

## Results

As stated in Materials and Methods, the MST-*k*NN agglomerative algorithm was applied to analyse the data set. As a basis for this algorithm a distance matrix using the Spearman rank correlation was used to calculate the distance among all respondents.

Using the distance between all the respondents, a complete weighted graph was built and the MST-kNN algorithm was able to compute its Minimum Spanning Tree and *k*-Nearest Neighbor proximity graphs. As previously explained, the value of *k* in the computation of the *k*-NN proximity graph can affect the size of the clusters, as the value of *k* influences whether the *k*-NN graph is more or less dense. For instance, when the value of *k* is set to 1, an edge that connects two respondents *x* and *y*, indicates that one of them, or both, are the most similar respondents in the entire data set and are therefore “nearest neighbors” of each other.

**Fig 2:**
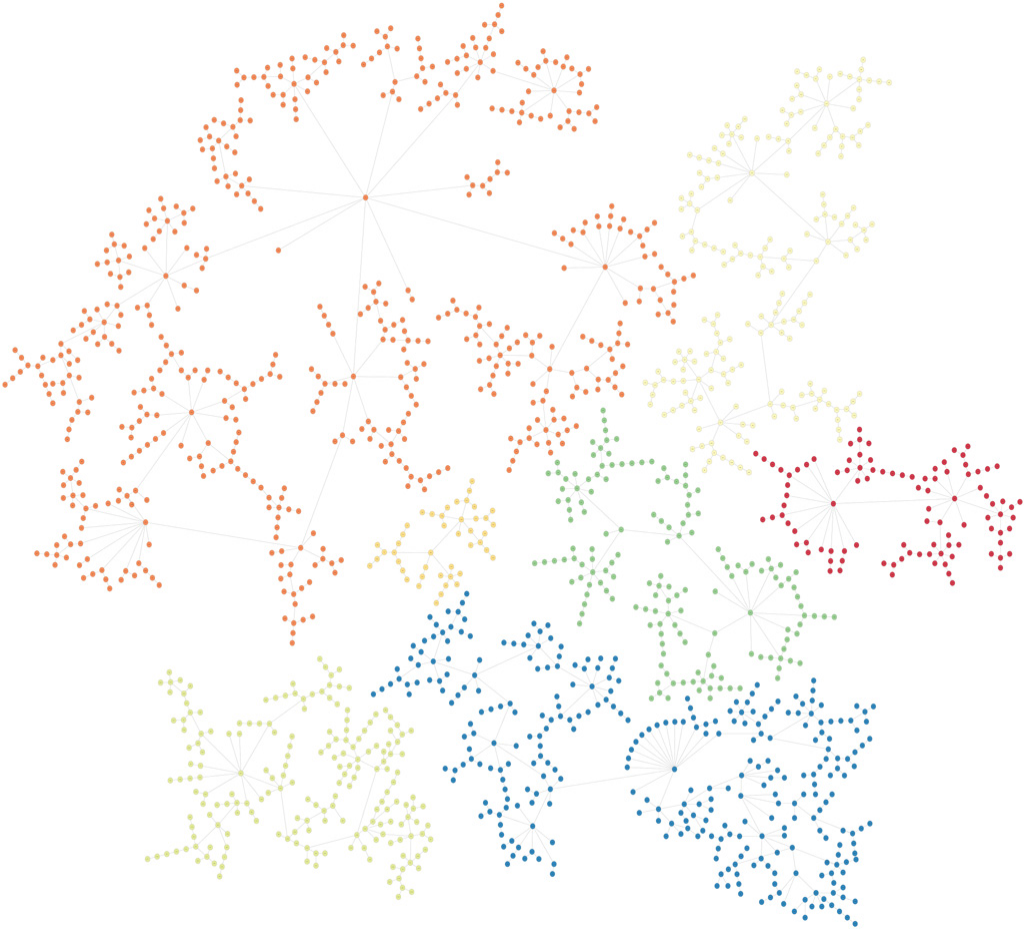
Results of the clustering method with *k* = 3. Seven clusters were found; Cluster 0 to Cluster 6. Clusters are of varying sizes with the largest cluster (Cluster 5) containing 556 respondents and the smallest cluster (Cluster 4) containing 45. Cluster 0 is shown in light yellow, Cluster 1 in green, Cluster 2 in light green, Cluster 3 in blue, Cluster 4 in light orange, Cluster 5 in orange and Cluster 6 in red.

Although results with the value of *k* = 1 show a natural bound between two respondents and perhaps the upper limit of the heterogeneity present in the data, it produces a large amount of very small clusters. In this study we attempt to identify clusters that can provide a basis for segmenting the population in terms of their trust and confidence in charities which can be used for targeted marketing and advertisement purposes. Such an exercise needs segments that are identifiable and viable to pursue from a marketing perspective. After computing various values for *k* = 1 and *k* = 2, we choose *k* = 3, a selection that produced a smaller number of larger clusters (seven in total). The outcome of the MST-kNN (with *k* = 3) clustering method produced clusters of relevant sizes as shown in Fig 2.

### Analysing the Clusters

As previously stated, in order to describe the clusters found by the MST-kNN clustering method, the CM1 score is employed. The selected ‘bottom’ and ‘top’ variables (or features) in terms of the CM1 scores for each cluster are presented in the Tables 1 and 2 respectively. Table 1 presents the bottom CM1 scores in ascending order with the distinctive features for each cluster highlighted. Table 2 presents the top CM1 scores in descending order with the most salient features highlighted for each cluster. The bottom features contain the most negatively ranked features while the top features contain the most positively ranked features for each cluster. In the context of this study, the bottom features highlight the questions with the lowest rates for a specific cluster compared to others, while the top features highlight the questions with the highest rates for a specific cluster compared to others. This is why these scores can be used in the analysis of each cluster to describe the respondents within each cluster. In the following section each cluster will be presented and described. After this, the cluster-specific models for involvement will be computed.

**Table 1:** Bottom features for each cluster (presented in ascending order of score).

Cluster 0	Cluster 1	Cluster 2	Cluster 3	Cluster 4	Cluster 5	Cluster 6
Q7B_7	**Q9_13**	**Q9_10**	**Q11_6_1**	Q9_15	**Q9_07**	Q11_1_1
Q7B_11	**Q9_14**	**Q9_02**	Q11_5_1	Q9_04	**Q9_05**	Q11_5_1
**Q7B_9**	**Q9_19**	Q7B_7	Q11_1_1	**Q9_16**	**Q9_06**	**Q11_4_1**
Q7B_6		Q7B_6	**Q11_3_1**	**Q9_21**		
**Q7B_5**		**Q9_03**	**Q11_2_1**	**Q9_24**		
**Q7B_10**		Q9_23	Q9_15	**Q7B_1**		
**Q7B_3**		Q7B_11	Q9_04	**Q9_25**		
				Q9_23		
				**Q7B_2**		
				**Q9_20**		
				**Q9_22**		

**Table 2:** Top features for each cluster (presented in descending order of score).

Cluster 0	Cluster 1	Cluster 2	Cluster 3	Cluster 4	Cluster 5	Cluster 6
Q9_21	Q9_07	Q9_07	Q9_02	Q11_5_1	**Q9_14**	**Q7B_4**
**Q9_23**	Q9_06	Q9_05	**Q9_10**	Q11_4_1	**Q9_13**	**Q7B_6**
Q9_20	Q9_05	Q9_06	**Q9_09**			Q7B_11
**Q9_24**	Q9_21	Q11_5_1	Q7B_7			Q7B_3
Q11_5_1	Q9_20	Q11_3_1	Q7B_11			Q9_02
Q9_06	Q11_5_1	Q11_6_1	**Q7A**			Q7B_7
Q11_3_1		Q9_21	**Q9_03**			
Q9_05		Q9_20	**Q9_19**			
**Q9_22**		Q11_1_1				
**Q9_25**		Q11_4_1				
Q11_6_1		Q11_2_1				
Q9_07						
Q11_1_1						
**Q9_04**						
**Q9_16**						
**Q9_15**						
Q11_4_1						
Q11_2_1						

#### Cluster 0—Non-institutionalist charity supporters

**Fig 3:**
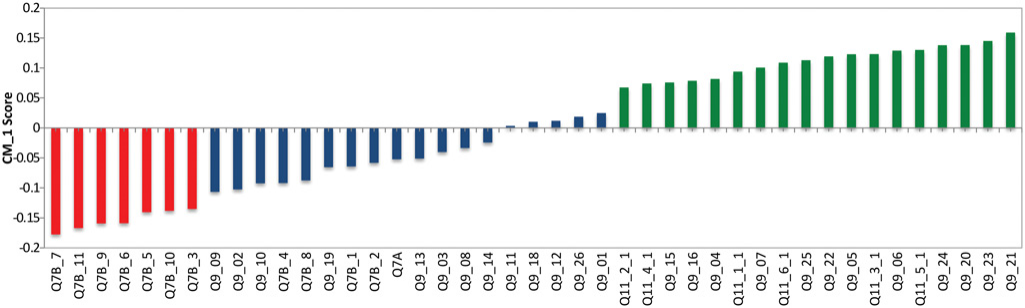
CM1 Scores of Cluster 0. The selected top and bottom features are shown in red and green respectively. As can be seen, these coloured features form a “shoulder” on either side of the ‘curve’ as they are characteristically higher or lower than the rest of the bars in this bar chart. The selected bottom and top “shoulders” are also presented in Tables 1 and 2.

Cluster 0, shown in light yellow in Fig 2, contains 195 of the respondents (13%) in this study and has 57% females and 43% males. The CM1 scores for the 43 features of Cluster 0, with the ‘bottom’ and ‘top’ features shown in red and green respectively are shown in Fig 3 as well as in Table 1. The question *‘How much trust and confidence do you have in the following institutions and organizations?’* (Q7B), which aims to rate the respondents’ trust and confidence levels in a variety of groups and institutions is shown to dominate amongst the bottom features of this cluster. The specific groups and institutions that form part of this cluster’s bottom features are:

- *Australian Taxation Office (ATO)* (Q7B_9),
- *Banks* (Q7B_5),
- *High Court* (Q7B_10) and;
- *Religious Organizations* (Q7B_3)

This result means that this cluster has a lower than average score for these variables when compared to all other respondents. Furthermore, these characteristics are exclusive (or distinctive) to this cluster meaning that no other cluster has these variables in its bottom CM1 score features. From this, we can see that these respondents have a lower level of trust in institutions when compared to other clusters. Although this is not the aim of this study, these characteristics could help charities and NFP institutions identify specific consumer groups and how to reach and build relationships with these consumers.

When analysing the ‘top’ CM1 scores for Cluster 0, that is, those scores which are on average higher for this cluster than all other respondents, the question *’When thinking about Australian charities, how much do you agree or disagree with the following?’* (Q9), is highly represented in the distinctive top CM1 scores. Specifically, the following statements are distinct to this cluster’s top CM1 scores:

- *I trust charities to ensure that a reasonable proportion of donations make it to the end cause* (Q9_23),
- *I trust charities to ensure that their fund raisers are ethical and honest* (Q9_24),
- *I trust charities to be well managed and efficient* (Q9_22),
- *I trust charities to act in the public interest* (Q9_25),
- *I trust charities that let the public know how they use their resources, including money from donations* (Q9_4),
- *I trust charities to make a positive difference to the cause that they are working for* (Q9_16) and;
- *I trust charities more if they are clear about how they are managed* (Q9_15)

These statements suggest that the respondents in this cluster trust charities, however, they are concerned with how the charity is managed and how charities manage their resources. These distinctive statements regarding trust, indicate that these respondents are more likely to trust (or support) charities and not-for-profit organisations as long as their “need” for information about the management of the charities is satisfied. Furthermore, from these results we can deduct that this cluster “carefully” supports charities rather than blindly following the crowd or trends.

#### Cluster 1—Resource allocation critics

**Fig 4:**
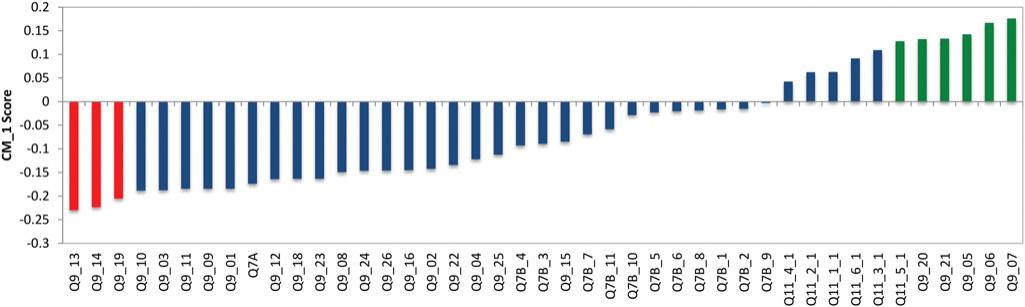
CM1 Scores of Cluster 1. The selected top and bottom features are shown in red and green respectively. As can be seen, these coloured features form a “shoulder” on either side of the ‘curve’ as they are characteristically higher or lower than the rest of the bars in this bar chart. The selected bottom and top “shoulders” are also presented in Tables 1 and 2.

Cluster 1 is shown in green in Fig 2 and has 157 respondents which makes up 10% of the respondents of the study. In Cluster 1, 55% are females and 45% males. The three ‘bottom’ and six ‘top’ CM1 scores for Cluster 1 are shown in red and green respectively in Fig 4 and presented in Tables 1 and 2. The bottom features consist of statements from the question: *‘When thinking about Australian charities, how much do you agree or disagree with the following?’* (Q9). Specifically, the following statements are the only bottom features for this cluster:

- *I trust charities that are well-known* (Q9_13),
- *I trust charities that have been established a long time* (Q9_14) and;
- *I trust charities with well-know supporters and patrons* (Q9_19)

These bottom CM1 score features are distinctive to Cluster 1 and can be interpreted as a relatively lower concern for charities’ “superficial reputation” for these individuals when compared to all other respondents. This means that they potentially care less about whether a charity or NFP organisation is well know, has been established a long time or whether the charity has celebrity supporters. The top CM1 scores of Cluster 1 explain these individuals further. Various statements from Q9 and one statement from Q11 are the top CM1 features. These statements are not exclusive to Cluster 1 and they are as follows:

- *Charities waste too much money* (Q9_07)
- *Charities spend too much of their funds on administration* (Q9_06)
- *Charities spend too much of their funds on salaries and staff benefits* (Q9_05)
- *I do not trust charities that pay sales people to raise funds* (Q9_21),
- *I do not trust charities that spend a lot of money on advertising* (Q9_20) and;
- *In the second column please rate how important it is to you that Australian charities provide this kind of information: The proportion of total funds spent on administrative costs* (Q11_5_1)

Although these top features are not exclusive to Cluster 1, there is a clear trend in these results as they are all centred around financial and managerial concerns. This cluster has lower than average scores in their concerns for charities’ and not-for-profit organisations’ superficial reputation, however, their care and concern for charities’ financial resource allocation seems to be a prominent characteristic of this cluster. The negative statements about financial concerns in the top CM1 score results indicate these individuals are critical on how charities manage their finances and less trust-worthy of charities and NFP’s that allocate resources to administrative and advertising costs. These characteristics show that the individuals in this cluster are quite critical, governance-aware, and feel negatively about the way they think charities allocate their financial resources.

#### Cluster 2—Information-seeking financial sceptics

**Fig 5:**
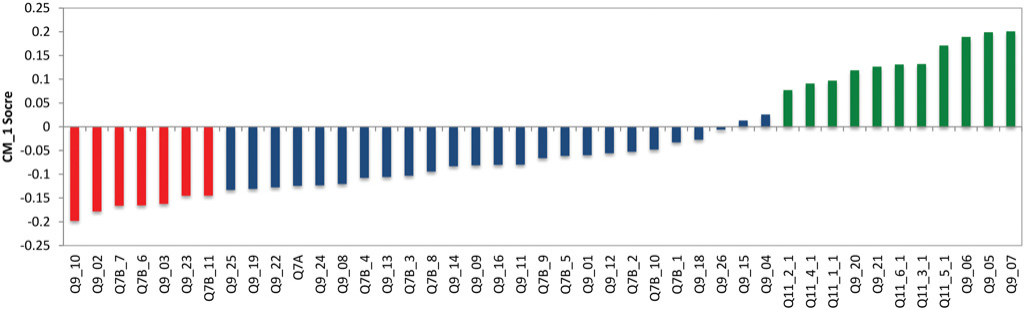
CM1 Scores of Cluster 2. The selected top and bottom features are shown in red and green respectively. As can be seen, these coloured features form a “shoulder” on either side of the ‘curve’ as they are characteristically higher or lower than the rest of the bars in this bar chart. The selected bottom and top “shoulders” are also presented in Tables 1 and 2.

Cluster 2, shown in light green in Fig 2, contains 192 respondents (12% of the total sample) with 60% females and 40% males. Fig 5 demonstrates the top and bottom CM1 scores for Cluster 2 with the 7 bottom and 11 top features shown in red and green respectively (as presented in Tables 1 and 2). Similar to Cluster 0 and Cluster 1, Q9: *‘When thinking about Australian charities, how much do you agree or disagree with the following?’* is highly represented for Cluster Two’s bottom CM1 scores and the distinct statements from this question for this cluster are:

- *I trust charities that provide services overseas* (Q9_10),
- *I feel confident donating to a charity even if I have not heard of them, if it is going to a good cause* (Q9_02) and
- *Charities are regulated and controlled to ensure that they are working for the public benefit* (Q9_03).

This means that this cluster has a lower than average score for these statements when compared to the others, which suggest that the charities’ reputation is a important factor for these individuals.

Looking at the top features, all the same statements from Q9 that were part of the top features for Cluster 1 are also part of the top features for Cluster 2 (Q9_7, Q9_05, Q9_06, Q9_21 and Q9_20). In addition to these, the top scores in Cluster 2 also include all statements from Q11 which states *“Some types of information that charities may provide are described below. Please provide the following ratings: In the first column, please rate how important it is to you that Australian charities provide this kind of information.”* The following statements form part of this question:

- *How charities use donations* (Q11_1_1)
- *Programs and services charities deliver* (Q11_2_1)
- *Charities’ fundraising costs* (Q11_3_1)
- *Impact of charities’ work* (Q11_4_1)
- *The proportion of total funds spent on administrative costs* (Q11_5_1)
- *The proportion of total funds spent on the charity’s work* (Q11_6_1)

These results indicate that this cluster has a greater concern with the charities’ and not-for-profit organisations’ information and financial resources. This cluster places a vital importance on the disclosure of information by charities and NFP organisations. This cluster wants to be informed and the way in which charities disclose (or withhold) any information or the way in which resources are allocated may influence their decision of supporting a charity.

#### Cluster 3—Non-questioning charity supporters

**Fig 6:**
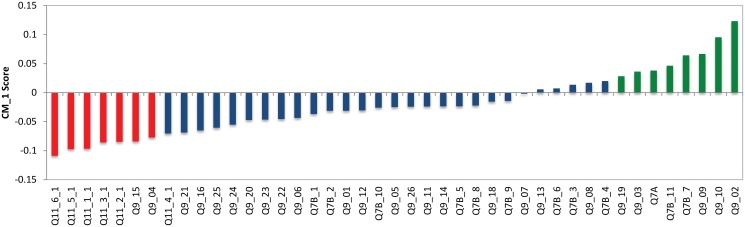
CM1 Scores of Cluster 3. The selected top and bottom features are shown in red and green respectively. As can be seen, these coloured features form a “shoulder” on either side of the ‘curve’ as they are characteristically higher or lower than the rest of the bars in this bar chart. The selected bottom and top “shoulders” are also presented in Tables 1 and 2.

Shown in blue in Fig 2, Cluster 3 has 317 respondents (20% of the total sample) with 61% females and 39% males. Fig 6 shows the 7 bottom and 8 top features for Cluster 3 in red and green respectively according Tables 1 and 2. Analysing the bottom features, the question: *“Some types of information that charities may provide are described below. Please provide the following ratings: In the first column, please rate how important it is to you that Australian charities provide this kind of information.”* (Q11) appears as the most negative ranked question for this cluster. The distinct features from this question for Cluster 3 as a bottom features include:

- *The proportion of total funds spent on the charity’s work* (Q11_1_6),
- *Charities’ fundaraising cost* (Q11_1_3) and
- *Programs and services the charities deliver* (Q11_1_2).

These features only appear as bottom features for this cluster and suggests that these individuals consider it to be less important that charities provide this kind of information compared to other clusters. Moreover, we can observe that these statements are related to financial information which suggests less care about the management of charities’ financial resources. This is quite contrary to Cluster 2 and Cluster 1 which place a great importance on the disclosure of this information.

When examining the “top” features, the question *How much trust and confidence do you have in Australian charities overall?* (Q7A) is observed as a distinct feature to this cluster and demonstrates a higher trust and confidence rate in Australian charities overall. Meaning that this cluster rates, on average, higher than other clusters in this question. Furthermore, the question *When thinking about Australian charities, how much do you agree or disagree with the following?* (Q9) presents the following distinct statements for this cluster:

- *I trust charities that provide services overseas* (Q9_10),
- *I trust big charities more than smaller ones* (Q9_09),
- *Charities are regulated and controlled to ensure that they are working for the public benefit* (Q9_03) and
- *I trust charities with well-known supporters and patrons* (Q9_19)

These statements indicate that these individuals are more likely to support large and well-known charities and are in general, charity supporters.

#### Cluster 4—Non-trusting sceptics

**Fig 7:**
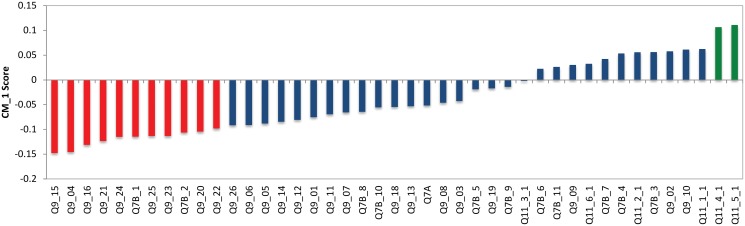
CM1 Scores of Cluster 4. The selected top and bottom features are shown in red and green respectively. As can be seen, these coloured features form a “shoulder” on either side of the ‘curve’ as they are characteristically higher or lower than the rest of the bars in this bar chart. The selected bottom and top “shoulders” are also presented in Tables 1 and 2.

Cluster 4 is the smallest cluster, shown in light orange in Fig 2 and includes 45 respondents (3% of the total sample). 47% are females and 53% are males. This cluster is the only one which presents a greater number of males than females. Fig 7 demonstrate the CM1 score for the 43 features for Cluster 4, with the 11 bottom and 2 top features shown in red and green respectively. The features from question *When thinking about Australian charities, how much do you agree or disagree with the following?* (Q9) dominate as a bottom feature. The individual statements from this question for Cluster 4 include:

- *I trust charities to make a positive difference to the cause that they are working for* (Q9_16),
- *I do not trust charities that pay sales people to raise funds* (Q9_21),
- *I trust charities to ensure that their fund raisers are ethical and honest* (Q9_24),
- *I trust charities to act in the public interest*(Q9_25),
- *I do not trust charities that spend a lot of money on advertising* (Q9_20) and,
- *I trust charities to be well managed and efficient* (Q9_22).

These statements suggest a lower concern about Australian charities’ activities. Moreover, the question *How much trust and confidence do you have in the following institutions and organizations?* (Q7B) also are individual features among the bottom feature and indicates a lower level of trust and confidence in *Doctors* (Q7B_1) and *Police* (Q7B_2).

When analysing the top features, the question *“Some types of information that charities may provide are described below. Please provide the following ratings: In the first column, please rate how important it is to you that Australian charities provide this kind of information.”* (Q11) appears as the most positive ranked question. Although this is not a exclusive characteristic for this cluster, this suggests a greater concern about the information provided by charities.

#### Cluster 5—Charity management believers

**Fig 8:**
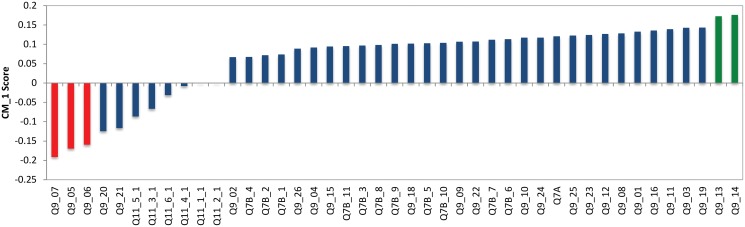
CM1 Scores of Cluster 5. The selected top and bottom features are shown in red and green respectively. As can be seen, these coloured features form a “shoulder” on either side of the ‘curve’ as they are characteristically higher or lower than the rest of the bars in this bar chart. The selected bottom and top “shoulders” are also presented in Tables 1 and 2.

Cluster 5 is the largest cluster with 556 respondents (36% of the total sample), shown in orange in Fig 2 and has 62% females and 38% males. Fig 8 shows the 3 bottom and 2 top features for Cluster 5 in red and green respectively.

The following distinct statements:

- *Charities waste too much money* (Q9_07),
- *Charities spend too much of their funds on salaries and staff benefits* (Q9_05),
- *Charities spend too much of their funds on administration* (Q9_06)

from question *“When thinking about Australian charities, how much do you agree or disagree with the following?”* (Q9) are shown as a bottom features for this cluster. It indicates that these respondents do not perceive wastefulness, as the others clusters, and they care less about the charities’ fund-raising. It is also observed that this is an opposite characteristic compared to the Cluster 0, 1 and 2. In addition, when analysing the top features, the statements:

- *I trust charities that have been established a long time* (Q9_14) and
- *I trust charities that are well-known* (Q9_13),

appear as distinct top features and indicate some concern about the charities’ reputation. Furthermore, these individuals are more likely to support well-known and long established charities.

#### Cluster 6—Institutionalist charity believers

**Fig 9:**
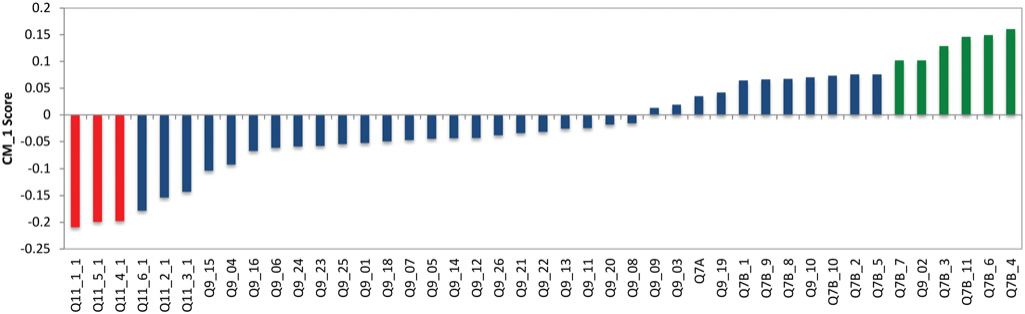
CM1 Scores of Cluster 6. The selected top and bottom features are shown in red and green respectively. As can be seen, these coloured features form a “shoulder” on either side of the ‘curve’ as they are characteristically higher or lower than the rest of the bars in this bar chart. The selected bottom and top “shoulders” are also presented in Tables 1 and 2.

Finally, shown in red in Fig 2, Cluster 6 contains 100 respondents (6% of the total sample) with 63% of female and 37% of males. Fig 9 shows the 3 bottom and 6 top features for Cluster 6 in red and green respectively. Looking at the bottom features, the question *“Some types of information that charities may provide are described below. Please provide the following ratings: In the first column, please rate how important it is to you that Australian charities provide this kind of information.”* (Q11) is the most ranked bottom feature with the following individual statement for this cluster:

- *Impact of charities’ work* (Q11_4_1),

This suggest a lower concern about the information provided by charities.

Among the top features the most ranked question is *How much trust and confidence do you have in the following institutions and organizations?* (Q7B), which indicates a higher trust and confidence level exclusively in the *News Media* (Q7B_4) and in their *Local council* (Q7B_6). Oppositely to Cluster 0, these respondents do trust institutions and are charity supporters as well.

### Cluster Partitioning Symbolic Regression Modeling Results

As described in Materials and Methods, the first experiment using symbolic regression analysis has been conducted in order to assess the results of the clustering method and the characterisation of the clusters given by the CM1 scores. Therefore, with the purpose of evaluating the classification accuracy, we have modelled a binary variable of the clusters in *Eureqa* and tried to find a model to predict which cluster a respondent is a member of as a function of all possible variables that formed CM1 scores for all clusters.

Each cluster has been modelled as the target variable of the symbolic regression process, meaning that each cluster had a ‘turn’ at having a binary variable value of 1 whilst all other clusters’ respondents had a value of 0 for the binary variable. As we are modelling a binary variable and as previously explained, a logistic function was introduced in concordance with *Eureqa’s* guidelines. Besides this function, the best simple models, (therefore, without multiplication, squares or the introduction of additional logistic functions) are selected for analysis here. The best models fitting these criteria are presented in Table 3.

**Table 3:** Best ‘simple’ logistic models for assessing cluster partitioning. For each model, the Fitness was guided by the Area Under the Curve value and is shown as well as the best model found by *Eureqa*.

Target Variable	Fitness	Model (Cluster = logistic(x))
Cluster 0	0.417	x = (Q11_5_1 + Q9_23—Q7B_9)
Cluster 1	0.293	x = (Q11_5_1 + Q9_06 + 0.9Q9_07 + 0.8 Q9_21 - 23 - Q9_19 - 2.5 Q9_13)
Cluster 2	0.324	x = (Q16 + Q19_05 + 8.41 Q10_4 + 1.1 Q9_10 - Q13 - Q19_07 - 10.4 Q10_8)
Cluster 3	0.600	x = (Q9_02 - 1.1 Q9_15)
Cluster 4	0.223	x = (Q11_4_1 + 0.3 Q11_5_1 - Q9_15 - 0.3 Q9_21)
Cluster 5	0.324	x = (Q9_13 + 2.02 Q9_14 - Q9_07 - Q9_05 - Q9_06)
Cluster 6	0.320	x = (Q7B_6 + 1.3 Q7B_4 + 0.4 Q9_02 - 1.48 - Q11_4_1 - 1.48 Q11_1_1)

The accuracy of these models depends on how well the model separates the respondents being tested into those belonging and those not belonging to the cluster in question. In this study, we have selected the option for *Eureqa* to optimise the Area Under Curve (AUC) in evaluating the quality of its mathematical models. A fitness value (error) of ‘zero’ thus represents a “perfect” model, in other words, a model that perfectly separates the respondents of the cluster in question from those of the other clusters. Therefore, the closer to zero the fitness values represented in Table 3 are, the better the models are to separate samples that belong to the cluster under consideration from those that belong to other clusters.

Five out of these seven models show fitness values lower than 0.350 with only one model having a fitness value higher than 0.50 (Cluster 3). More complex models were found by *Eureqa* with fitness (error) values much closer to zero, however, we have chosen to present those ‘simple’ models for ease of interpretation. The models have been compared against the CM1 scores in Tables 1 and 2 and as can be inspected, all variables found to have a positive relationship with the target variable of the cluster match that particular clusters’ top CM1 score features and conversely, all variables found to have a negative relationship with the target cluster match that cluster’s bottom CM1 score features. For some clusters almost all, or all (like in the case of Cluster 5), of the CM1 score features previously discussed are used in the model to predict respondents’ membership to that cluster and perfectly match to be either a positive relationship (from top CM1 score features) or a negative relationship (from bottom CM1 score features).

As stated, in the more complex models, more accurate error measures were found which show an even more accurate prediction of cluster membership for each of the clusters which satisfies our assessment of the cluster partitioning and description using the CM1 score.

### Cluster-specific Models of Involvement Symbolic Regression Modeling Results

Here we present and discuss the results of the symbolic regression process to predict ‘Low’ or ‘High’ Involvement of the respondents in each of the clusters.

Again, the introduction of a logistic function was implemented in order to deal with the binary variable. Other than this, ‘simple’ building blocks were again used. Subsequently, we have analysed the models found by Eureqa and have selected the best-fitting model in terms of AUC for each cluster that, within its logistic function, simply used linear regression functions. This gives us varied results for each of the clusters with *Eureqa* finding better-fitting models for some clusters than others. Nonetheless, these models provide us with useful information which can be used to interpret the consumers within each cluster as well as inform the NFP sector of more detailed implications regarding each of the clusters found in this study. The involvement class models selected for analysis for each cluster are presented in Table 4 as well as their corresponding fitness values.

**Table 4:** Best ‘simple’ logistic models for Involvement Class. For each cluster, the Fitness was guided by the Area Under the Curve value is shown as well as the best model found by *Eureqa*.

Cluster	Fitness	Model (QCLASS = logistic(x))
0: Non-institutionalist charity supporters	0.793	x = (Q19_14 - Q9_20)
1: Resource allocation critics	0.321	x = (Q7B_9 + Q9_02 + Q9_11 + Q16 - Q9_20 - 2.2 Q9_09 - 11.4 Q10_8)
2: Information-seeking financial sceptics	0.296	x = (Q11_5_1 + Q16 +12.4 Q10_4 +1.6Q9_10 -Q19_07 -Q9_05*Q13 -93.3Q10_8)
3: Non-questioning charity supporters	0.850	x = (Q7B_3 - 14;Q7B_2)
4: Non-trusting sceptics	0.331	x = (84.1Q10_7 - Q9_25)
5: Charity management believers	0.881	x = (Q9_16 - Q13)
6: Institutionalist charity believers	0.436	x = (2.5 Q9_15 - Q11_1_1 - Q19_07)

#### Cluster 0—Non-institutionalist charity supporters

As shown in Table 4, a simple model with only two variables satisfied the criteria outlined in the previous section. One variable with a positive relationship to predict involvement and one with a negative relationship. Interestingly, a variable from Question 19 entered the model for Cluster 0 which has not occurred in a model for any other cluster. This question asked respondents what types of information they would be interested in (on a scale of 1 to 10) to look up in a national registry for charities and NFP organisations, if this would be available. Specifically, Q19_14 relates to *“The names and positions of responsible people”*, meaning that people who are interested in doing this in Cluster 0 are more likely to be highly involved with charities. The other variable, Q9_20 relates to how respondents, on a scale of 1 to 10 agree with the statement *“I don’t trust charities that spend a lot of money on advertising”*. Although this is a negative statement about charities, in this cluster, it is negatively related with high involvement in charities. Meaning that respondents who answered lower on this scale are *more* likely to be in the high involvement class than those respondents who agreed with this statement more strongly.

#### Cluster 1—Resource allocation critics

In the *Eureqa* model to predict involvement for Cluster One there are four variables that positively influence involvement and three variables that negatively influence involvement as can be seen in Table 4. Specifically, Q7B_9 relates to a respondents’ trust in the Australian Taxation Office (ATO) meaning that those respondents in this cluster with higher levels of trust in the ATO are more likely to be highly involved with charities and show high involvement charitable behaviours. Furthermore, Q9_02 and Q9_11 also have a positive relationship with involvement for Cluster One and relate to a person’s confidence in donating to a charity they have not previously heard of before and their trust in charities that provide services in Australia, respectively. The fourth variable to have a positive effect on respondent’s involvement level is Q16 which asks respondents on a scale or 0 to 10, how much do they know about the Australian Charities and Not-for-profits Commission (ACNC). This means that those people who feel like they know more about the ACNC, are more likely to be highly involved with charities and NFP’s.

Looking at those variables in a negative relationship with involvement for this cluster we gain further insights. Q9_20 states *“I don’t trust charities that spend a lot of money on advertising”* and respondents had to answer on a scale from 0 to 10 whether they agreed with this statement. Q9_09 also has a negative relationship with involvement and asks respondents again on a scale of 0 to 10 to agree with a statement; *“I trust big charities more than smaller ones”*. Finally, Q10_8 has a strong negative impact on this cluster’s involvement which is a binary variable and equalled to one when a respondent had never done any activity such as finding out further information about the cluster or checked to see if it was a genuine charities.

#### Cluster 2—Information-seeking financial sceptics

For cluster two a fairly complex model was found for involvement class. There are four variables with a positive relationship and three variables with a negative relationship with involvement class. Firstly, Q16 is positively related to involvement class and asked respondents how much they feel they know about the ACNC (on a scale of 1 to 10). Next, Q19_05 is also positively related to involvement class. Question 19 overall relates to the types of information consumers may be interested in accessing from a national governing body of the NFP sector. Specifically, Q19_05 relates to the type of charity (e.g. welfare, education and training, accommodation, disability, children, etc.). This means that those respondents who answered they are likely to look for this type of information, are more likely to be part of the high involvement class. Next, the variable Q10_4 has a strong positive effect in this model and is a binary variable whether respondents had previously given to a charity they hadn’t heard of. The fourth positive variable in the model for Cluster 2 is Q9_10 which rates on a scale (1–10) how much respondents agree with the statement: *“I trust charities that provide services overseas”*. This means that respondents who more strongly agree with this statement are more likely to be in the high involvement class.

The first negatively related variable is Q13 which is a yes/no question (i.e. a binary variable) and stated: *“To the best of your knowledge, is there an organisation or agency that is responsible for watching over the activities of Australian charities?”* where a ‘Yes’ answer constituted a 1 and a ‘No’ answer is given the weight equal to 2. This configuration of the binary variable means a ‘No’ answer to this question exerts a stronger negative impact in the equation whereas a ‘Yes’ answer obviously has a lesser negative impact. This means that those respondents who answered ‘No’ are in fact more likely to be in the high involvement class than those respondents who did know there was a governing “watchdog” organisation for the NFP sector. The next negatively impacting variable is Q19_07 which asks respondents (on a scale of 1 to 10) whether they would be interested in where the charity operates (e.g. states in Australia or overseas) which means that those respondents who answered higher on this scale, are less likely to be highly involved. Lastly, Q10_8 is a negatively impacting variable in the model for Cluster 2. Question 10 stated *“When you have given money to a charity, have you ever done any of the following?”* with several options to say yes/no. Therefore, this is another binary variable. What is interesting is that Q10_8 states *“None of the above”*. This means that Cluster 2’s respondents who had not proceeded to check the charity’s status, or claimed a tax refund are less likely to be in the high involvement class.

#### Cluster 3—Non-questioning charity supporters

For cluster 3 only a simple model satisfied the criteria we set for analysing involvement class models. Unfortunately, the fitness value is not of very good quality (0.85) as shown in Table 4, however, this model is included for completeness and used to motivate an initial interpretation. Should charities wish to generate targeted marketing strategies for each of these clusters, models of any complexity levels could be used. Furthermore, it is important to add that in most more complex models, the two variables in this model are still included. The fact that *Eureqa* has found these two variables first and they are still included in almost all other models means that these variables are perhaps of considerable importance in predicting involvement class in this cluster. One positive and one negatively impacting variable make up the simple model for the non-questioning charity supporters to predict involvement class. Firstly, Q7B_7 relates to respondents’ trust in the Federal Parliament (on a scale of 1 to 10) and secondly, Q7B_2 to respondents’ trust in the police. Remarkably, that means that for this cluster, those respondents with higher levels of trust in the Federal Parliament and lower levels of trust in the police, are more likely to be in the high involvement class.

#### Cluster 4—Non-trusting sceptics

As can be seen in Table 4, Cluster 4 also has a model with only two variables. However, the model for cluster four has a fitness value of 0.321, which means it is of reasonable accuracy. Again, like for Cluster 3 we have a case of one positive impact and one negative. The strongly positively impacting variable (with a coefficient of 84.1) is Q10_7 which was a yes/no question whether respondents had previously checked to see if the charity was registered after they had given money to that charity. Considering Cluster 4 has been labelled as the non-trusting sceptics” it is logical that respondents in this cluster would like to check further information and are more likely to be highly involved if they do so (provided that their information need is satisfied). This strong positive impact is offset by the negative impact of Q9_25 which rates on a 1−10 scale whether respondents “trust charities to act in the public interest”. Considering this cluster is the ‘non-trusting sceptic’ cluster, finding these impacts on involvement class are interesting even though it is only a small cluster.

#### Cluster 5—Charity management believers

For cluster five we again have a small model with a non-desirable fitness value (0.881). However, as stated, we use this as the initial interpretation of high involvement for the cluster and these variables are included in almost all other (more complex) models. The first variable, with a positive impact on involvement class is Q9_16 which rates how much respondents agree (on the 1–10 scale) with the statement: *“I trust charities to make a positive difference to the cause that they are working for”*. This means that for the ‘charity management believers‘ they are even more likely to be highly involved with charities if they rate higher on this statement. Conversely, Q13 is negatively related with involvement class which, as stated, asked respondent of their knowledge of a national ‘watchdog’ for the NFP sector. Again, the same applies here as for cluster two due to the peculiar configuration of this binary variable. A ‘Yes’ answer again for this cluster means a lesser negative impact and therefore means that those Cluster 5 respondents who previously were aware of a national organisation for the NFP sector are actually more likely to be in the high involvement class.

#### Cluster 6—Institutionalist charity believers

For cluster six, *Eureqa* has found a model with one positively influencing variable and two negative ones. The positive variable, strengthened with a coefficient of 2.5, is Q9_15 which rates on a 1–10 scale how much respondents agreed with: *“I trust charities more if they are clear about how they are managed”*. This means that those respondents who agree with this statement more strongly, are more likely to be in the high involvement class. The first negative variable is Q11_1_1 which relates to the importance respondents place on charities providing information about how they use donations (on a 1–10 scale). For the institutionalist charity believers it means that as as respondents in this cluster agreed more strongly to this, they are in fact, less likely to be highly involved. Finally, Q19_07 is also negatively related to involvement class in this cluster. As stated, question 19 asked respondents how likely it is that they seek certain types of information from the ACNC. Specifically, Q19_07 relates to *“Where the charity operates”*. This corresponds with the same variable in cluster two wherevQ19_07 also had a negative impact.

The specific findings for each of the clusters outlined in this section are further elaborated on in the final section including the discussion. A general overview of our study is presented followed by some specific implications for charity and NFP managers. Furthermore, we highlight the contribution of this work, as well as our limitation and future research recommendations.

## Discussion

### Significance and Contribution

The results from this study provide the charity and NFP marketing literature with various interesting implications. Firstly, this study is among the first to utilise the novel clustering methodology of the MST-kNN method in a context of marketing and, specifically, it is the first application in the field of charities and not-for-profit organisations. Secondly, the application of the CM1 score to obtain easily interpretable salient features which better characterise each segment, provides literature and practice with a new approach to understand, investigate and describe clusters or segments more easily without a priori assumptions. As stated, this data-driven approach to consumer segmentation has become almost crucial for charities. The sector needs to move towards private-industry type marketing strategies in order to ‘survive’ and continue maintaining their levels of private donors’ support. Our selection of the Australian sector was motivated by the fact that it is experiencing a multifaceted challenge. On one side, there exists a large number of charitable organizations relative to the population size and, on the other, there is the perceived notion that they already have enough public funding and subsequently do not greatly depend on private donations.

Furthermore, the method we have used in this study is easily transferable to different data sets and various situations. A charity or not-for-profit institution could easily apply this clustering method to its existing donor database in order to identify groups for personalisation within their collected information. Likewise, this method could also be used to examine a wider segment of the national population, possibly a certain geographic area close to a particular institution, to identify those segments of the population worth pursuing in their marketing campaigns. In doing so, this method helps charities avoid the use of wasteful mass-marketing campaigns and instead, personalise their advertising and communication efforts to be more suited to each donor segment.

### Cluster-specific strategies for the NFP sector

The clusters identified in this study provide an initial insight into the specific donor segments of the Australian market. For each cluster there are specific actions charity managers could undertake in order to drive these consumers (possible donors) from ‘low involvement’ to ‘high involvement’. The motivation for these actions can directly be taken from the symbolic regression result findings.

We start with Cluster 0, the non-institutionalist charity supporters. Although, their CM1 scores indicated that these respondents tend to be ‘charity supporters’ there is always room to increase donors’ support of charities. Therefore we turn to the models found from *Eureqa*. For this cluster, charity managers could make the names and positions of the responsible people within the organisation more accessible to the public (or to the donors) in order to drive involvement from this cluster. Furthermore, as we identify these respondents as ‘non-institutionalists’, it could work in a charity’s favour to avoid reaching these consumers through partnerships with large institutions as their levels of trust in institutions are lower than all other respondents. If a charity is effective in providing information on their operations and management, the ‘non-institutionalist charity supporters would be an attractive target market.

Looking at Cluster 1, the ‘resource allocation critics’, the aspects that hinder involvement for this cluster are in line with their CM1 score description. These consumers do not like charities that have high levels of “perceived wastefulness”, that is, charities who spend a lot of funds on administrative or advertising costs. What drives involvement for this cluster is their knowledge if the ACNC and the fact that they like charities that provide services in Australia (i.e. in their own country). An interesting observation about this cluster is that donating to a charity they had not previously heard of before, drives involvement in this cluster. For the NFP sector this means that these respondents may be more likely than others to make a donation to a charity they had not previously heard of before. For charities to target this cluster they need to remember to be mindful about where their funds and resources go as well as the reporting of where their resources go. Furthermore, informing consumers about the ACNC could also help drive involvement for the ‘resource allocation critics’.

For Cluster 2, different tactics apply, should charity managers wish to target these consumers. These ‘information-seeking financial sceptics’ are more likely to be highly involved with a charity when they have greater knowledge of a national NFP sector ‘watchdog’ organisation. This means that charities could educate consumers in their respective possible donor base about what type of organisation they report to and what organisation(s) there are to regulate the charity and NFP sector. As was argued in the Introduction, trust and confidence in charities are closely linked. For the respondents in this cluster this possibly means that with a greater knowledge of a national governing body for the NFP sector, they trust charities more, and are subsequently more likely to be involved with charities in terms of donating time or money. As their description suggest, information about the charity or NFP is important to them. Furthermore, whether cluster one respondents had previously heard of a charity before and knowing what type of charity it is also drives their involvement. This means that with basic communication messages, charities can inform consumers of this information and drive higher levels of involvement from cluster two.

Due to the small model found for Clusters 3, 4 and 5, only limited inferences can be made. For Cluster 3, the ‘non-questioning charity supporters’, the simple symbolic regression model helps charities to better define this cluster. For example, knowing that trust in the federal parliament impacts involvement for these consumers means that charities could follow trends in the population’s trust in the government and act accordingly. Furthermore, interestingly, trust in police negatively impacts involvement in Cluster 3 meaning that to target these consumers, charities should avoid partnerships with the police force or having police officer spokespersons. Cluster 4, the ‘non-trusting sceptics’ may be hard to convince, however, several actions may help to convert these consumers to possible donors. When these consumers checked whether charities were registered (after donating money) they were more likely to have higher levels of involvement. This signals to charities that it is of high importance to ensure they are registered. Cluster 5, the ‘charity management believers’, is most likely quite opposite to Cluster 4. These consumers do trust charities to make a positive difference towards the cause they are working for, and the more they are convinced of this, the more they are likely to be highly involved. This makes Cluster 4 an attractive group of potential donors to target. Charities management’s task is to then keep these supporters up to date of regular progressions in the work they are doing and the positive effects of their efforts. Furthermore, as with Cluster 2, these respondents are more likely to be highly involved when they know more about a national organisation ‘watchdog’ of the charities and NFPs sector. Again, charities can pass on the information of such an organisation which could improve trust and involvement levels from donors.

Cluster 6, the ‘institutionalist charity believers’, are more likely to display high involvement behaviours towards charities if the charity is clear about how they are managed. For charity and NFP management this simply means to be transparent in the way they operate, how they are organised, who is responsible and other information regarding the management of the charity. This information could be provided on the charity’s web page, their news letters, or additional actions could be taken such as the provision of contact details which donors can use to request further information. The negatively related variables for this cluster are interesting. They are in concordance with their label as ‘institutionalist charity believers’ since these consumers do not place importance on the charity providing information on the use of donations. In fact, this variable negatively impacts involvement. Furthermore, these respondents do not become more involved with the charity as a result of finding out where it operates meaning that this kind of information is not of great importance to the members of this cluster. Again, this means that Cluster 6 provides an attractive market segment for charities to target.

Overall, several trends and behaviours of potential donors have been highlighted by our study. We have found some clusters in which charities and NFP’s are more likely to be successful in obtaining donations than in others. For instance, for a new charity with limited resources, it would not be advisable to target Cluster 4, the ‘non-trusting sceptics’ as they would likely be a lot less successful than with consumers part of Clusters 0, 5 or 6; the ‘non-institutionalist charity supporters’, the ‘charity management believers’ and the ‘institutionalist charity believers’, respectively. One interesting trend found across almost all clusters is the donor’s concerns with the financial resource allocation within charities. Even in the involvement models for those clusters that are charity ‘supporters’ or ‘believers’, financial concerns surface. This means that the findings in this study are in concordance with Gneezy et al.’s [11] comprehensive study on avoiding overhead aversion. As stated, they found that donations increased significantly if seed funding for overheads was already covered and potential donors were informed of this. The message to the NFP sector from this general trend is that charities and NFP organisations need to, no matter which market segment they are targeting, be transparent about their resource allocation, avoid allocating too many funds to advertising and administration activities (where possible) and be open to their donors about their operations and management.

### Limitations and future research

Although this study provides literature and charity and not-for-profit marketing managers several implications and contributions, there are some limitations. Firstly, the data set used came with some limitations. As it was an online administered questionnaire, the system was able to direct respondents to various parts of the survey as well as skip questions that did not apply to some of them. This left several items uncompleted where some or the majority of respondents did not answer all questions. In order to be able to still use the entire dataset, we omitted those questions (variables) that not every respondent answered. This reduced the number of variables we were able to use in our clustering method and subsequent cluster analysis.

Another limitation we have found with the dataset is the way the ‘Involvement Class’ is constructed. The ACNC assigned respondents to either the ‘low involvement class’ or the ‘high involvement class’ based on their responses in question 3. There were several questions that, if respondents answered ‘yes’, it would automatically classify them as ‘high involvement’. For instance, whether they had made regular monthly donations to a charity or sponsored an animal or a child via a charity. Although we do not argue that it is logical to assume certain activities are of a higher involvement level with a charity than others, we do argue that a respondent could feel like they are highly involved with a charity even though they had not answered ‘yes’ to these questions. One example of an activity classified as ‘low involvement’ by the ACNC is *“Sold raffle tickets or conducted other fundraising on behalf of a charity”*. We note, that if an individual supports a charity by selling raffle tickets once, we resonate with this claim, however, if a person spends a considerable amount of time doing so (for example, several days a week) it could also be reasonable to assume that this person is ‘highly involved’ with the charity they are supporting. For our study this means that the results found to define the involvement class could in the future be better defined if a better framework for classifying the ‘Involvement Class’ is designed. Instead of merging activity types into the dichotomy provided by ‘Low/High Involvement’ an alternative would be to seek mathematical models that could predict each one of the activities separately.

The last limitation we highlight is a conscious “self-imposed” limitation during the analysis of the symbolic regression modeling. As stated, we only selected those models for analysis that included ‘simple’ functions used by *Eureqa* (the argument for the logistic model is a linear function). We have also explained that more complex models have found better results in terms of the Area-Under-the-Curve, which means that in future studies more effort could be exerted in analysing these findings. Our aim in this study was to provide the literature and the NFP sector with an easily-interpretable method for segmentation and subsequent analysis. It is for this reason we have imposed this limitation and suggest future research directions to explore this path further.

Furthermore, future research could consider a mixed-method approach utilising a qualitative method such as in [2] combined with a quantitative method as outlined in this study for greater depth of results and to provide a more detailed analysis of each cluster or group of consumers. For example, in their qualitative research, Polonsky, Shelley and Voola [2] state that, “the strength of the stimulus related to personalized appeals and may create a link between the individual and the cause, which would increase donor’s support” [p.74].

Another future research suggestion is to conduct another study of a similar kind with a different questionnaire tool. In the current study, no questions about either actual monetary donations or time spent volunteering were included. These questions could help to more accurately gauge consumers’ behaviours and their attitudes towards charities and, therefore, help charities to understand their donor base even better by considering their individualities.

Future research could also extend the study across multiple countries and/or cultures in order to find more generalisable results which can then be applied in more countries and areas.

As stated, we have only used the best most simple *Eureqa* models. In the future, a new way could be constructed to automatically analyse all results found by *Eureqa’s* symbolic regression modelling method to assist the user of the clustering results in interpreting their findings. In this process, other Artificial Intelligence and Machine Learning methodologies could be employed to truly find the best underlying structure in the data. *Eureqa* employs a genetic programming technology which, combined with other types of algorithms to “learn from the data”, would lead to a new generation of more accurate models which, in turn, could provide more refined donor behaviour models.

Taking this study even further, the novel concept of “functional construct” as introduced in an earlier publication [24], could be applied in the context of NFP sector research. For instance, the data in this study comprised of separate questions, or “items”, which could be analysed by a process as outlined in de Vries et al. [24] to find functional constructs for further behavioural modelling. This would provide literature and the charity and NFP sector with an even deeper understanding of the relationship that exists between consumers’ attitudes, trust and confidence in and the behaviours such as donating they actually display towards charities. These constructs could even form the basis for other methods such as Structural Equation Modelling (SEM) as in [26] to find the structural relationships between ‘constructs’ of items. In a study investigating the charity and NFP sector in particular, a SEM could investigate the relationships between trust and donating behaviour or confidence and trust in a charity or the relationship between trust and volunteering.

These future research directions are bountiful and based on the results in this study we highly recommend the charity and NFP sector to continue their trend in this way to become more successful, less wasteful and ultimately, serve their true cause better and more efficiently. The clustering method in this study can lead to a data-driven marketing strategy as it allows charities to segment their donor market and then investigate their salient features with a relatively simple method that eases interpretation (the CM1 score). The technique is also highly scalable and can easily be applied to problems involving several millions of consumers [16]. Obviously, such large datasets are unlikely to come from questionnaire-based studies but with the increasing use of online communication channels, this is a realistic scenario and a research area worth pursuing.

The findings related to the cluster analysis collectively provide guidance and a better understanding of a possible donor base for the NFP and charity sector. If charities take actions to drive donor involvement with a data-driven approach, higher levels of monetary donations and an increase in volunteering efforts will ensue. By following for-profit organisations and businesses in being more informed about the environment and market they operate in, charities can hope to thrive, be more successful in obtaining funds and ultimately, achieve greater success in the causes they are working towards.

## References

[1] Shelley L, Polonsky J. Do charitable causes need to segment their current donor base on demographic factors?: An Australian examination. International Journal of Nonprofit and Voluntary Sector Marketing. 2002;7(1):19–29. 10.1002/nvsm.164

[2] Polonsky J, Shelley L, Voola R. An Examination of Helping BehaviorSome Evidence from Australia. Journal of Nonprofit & Public Sector Marketing. 2002;10(2):67–82. 10.1300/J054v10n02_04

[3] Sargeant A, Lee S. Donor Trust and Relationship Commitment in the U.K. Charity Sector: The Impact on Behavior. Nonprofit Volunt Sect Q. 2004;33(2):185–202. 10.1177/0899764004263321

[4] Schlegelmilch B. Targeting of Fund-raising Appeals How to Identify Donors. Eur J Mark. 1988;22(1):31–40. 10.1108/EUM0000000005265

[5] Yavas U, Riecken G, Parameswaran R. Personality, organization-specific attitude, and socioeconomic correlates of charity giving behavior. Journal of the Academy of Marketing Science. 1981;9(1–2):52–65. 10.1007/BF02723565

[6] Menp K. Clustering the consumers on the basis of their perceptions of the Internet banking services. Internet Research. 2006;16(3):304–322. 10.1108/10662240610673718

[7] Smith W. Product Differentiation and Market Segmentation as Alternative Marketing Strategies. J Mark. 1956;21(1):3–8. 10.2307/1247695

[8] Srnka K, Grohs R, Eckler I. Increasing Fundraising Efficiency by Segmenting Donors. Australasian Marketing Journal (AMJ). 2003;11(1):70–86. 10.1016/S1441-3582(03)70119-0

[9] Bekkers R, Bowman W. The Relationship Between Confidence in Charitable Organizations and Volunteering Revisited. Nonprofit Volunt Sect Q. 2009;38(5):884–897. 10.1177/0899764008324516

[10] Sargeant A, Lee S. Trust and relationship commitment in the United Kingdom voluntary sector: Determinants of donor behavior. Psychology and Marketing. 2004;21(8):613–635. 10.1002/mar.20021

[11] Gneezy U, Keenan E, Gneezy A. Avoiding overhead aversion in charity. Science. 2014;346(6209):632–635. 10.1126/science.1253932 25359974

[12] Inostroza-Ponta, M, Mendes, A, Berretta, R, Moscato, P. An Integrated QAP-Based Approach to Visualize Patterns of Gene Expression Similarity. In: Randall, M, Abbass, HA, Wiles, J, editors. Progress in Artificial Life, Third Australian Conference, ACAL 2007, Gold Coast, Australia, December 4-6, 2007, Proceedings. vol. 4828 of Lecture Notes in Computer Science. Springer; 2007. p. 156–167.

[13] Inostroza-Ponta M, Berretta R, Moscato P. QAPgrid: A two level QAP-based approach for large-scale data analysis and visualization. PLOS ONE. 2011;6(1):e14468 10.1371/journal.pone.0014468 21267077 PMC3022583

[14] Clark M, Johnston R, Inostroza-Ponta M, Fox A, Fortini E, Moscato P, et al Genome-wide analysis of long noncoding RNA stability. Genome res. 2012;22(5):885–898. 10.1101/gr.131037.111 22406755 PMC3337434

[15] Arefin, A, Riveros, C, Berretta, R, Moscato, P. kNN-MST-Agglomerative: A fast and scalable graph-based data clustering approach on gpu. In: Computer Science & Education (ICCSE), 2012 7th International Conference on. IEEE; 2012. p. 585–590.

[16] Arefin A, Mathieson L, Johnstone D, Berretta R, Moscato P. Unveiling clusters of RNA transcript pairs associated with markers of Alzheimers disease progression. PLOS ONE. 2012;7(9):e45535 10.1371/journal.pone.0045535 23029078 PMC3448659

[17] Herlocker, J, Konstan, J, Borchers, A, Riedl, J. An algorithmic framework for performing collaborative filtering. In: Proceedings of the 22nd annual international ACM SIGIR conference on Research and development in information retrieval. ACM; 1999. p. 230–237.

[18] Blattberg R, Do K, Scott N. Database Marketing: Analyzing and Managing Customers. 1st ed. 1 Auflage New York: Springer Verlag; 2008.

[19] Marsden J, Budden D, Craig H, Moscato P. Language Individuation and Marker Words: Shakespeare and His Maxwell’s Demon. PLOS ONE. 2013;8(6):e66813 10.1371/journal.pone.0066813 23826143 PMC3694980

[20] Filiou M, Arefin A, Moscato P, Graeber M. ‘Neuroinflammation differs categorically from inflammation: transcriptomes of Alzheimer’s disease, Parkinson’s disease, schizophrenia and inflammatory diseases compared. Neurogenetics. 2014;15(3):201–212. 10.1007/s10048-014-0409-x 24928144

[21] Aviad B, Roy G. A decision support method, based on bounded rationality concepts, to reveal feature saliency in clustering problem. Decis Supp Syst. 2012;54(1):292–303. 10.1016/j.dss.2012.05.037

[22] Smits G, Kotanchek M. Pareto-front exploitation in symbolic regression. In: Genetic programming theory and practice II. U.S.: Springer; 2005 p. 283–299.

[23] Huynh-Thu V, Irrthum A, Wehenkel L, Geurts P. Inferring Regulatory Networks from Expression Data Using Tree-Based Methods. PLOS ONE. 2010;5(9):e12776 10.1371/journal.pone.0012776 20927193 PMC2946910

[24] de Vries N, Carlson J, Moscato P. A Data-Driven Approach to Reverse Engineering Customer Engagement Models: Towards Functional Constructs. PLOS ONE. 2014;9(7):e102768 10.1371/journal.pone.0102768 25036766 PMC4103885

[25] Nutonian. Eureqa Pro Desktop - Academic. Nutonian Inc.; 2014.

[26] de Vries N, Carlson J. Examining the drivers and brand performance implications of customer engagement with brands in the social media environment. Journal of Brand Management. 2014;21(6):495 –515. 10.1057/bm.2014.18

